# 

**Published:** 1911-02-18

**Authors:** 


Telegraphic Address: Ospedale, London.

THE MODERN MEDICAL AND INSTITUTIONAL JOURNAL.

Telephone Number: 2734 Gerrard.

NEW SERIES. No. 211, Vol. VIII.

No. 1283, Vol. XLIX.

SATURDAY, FEB. 18TH, 1911.

PRICE THREEPENCE

